# Traditional Chinese medicine use in patients with oral cancer: A retrospective longitudinal cohort study in Taiwan

**DOI:** 10.1097/MD.0000000000030716

**Published:** 2022-09-23

**Authors:** Eyal Ben-Arie, Bernice Lottering, Chanya Inprasit, Hei-Tung Yip, Wen-Chao Ho, Gil Ton, Yu-Chen Lee, Pei-Yu Kao

**Affiliations:** a Graduate Institute of Acupuncture Science, China Medical University, Taichung, Taiwan; b Suphanburi Campus Establishment Project, Kasetsart University, Suphan Buri, Thailand; c Management Office for Health Data, China Medical University Hospital, Taichung, Taiwan; d College of Medicine, China Medical University, Taichung, Taiwan; e Department of Public Health, China Medical University, Taichung, Taiwan; f Department of Acupuncture, China Medical University Hospital, Taichung, Taiwan; g Chinese Medicine Research Center, China Medical University, Taichung, Taiwan; h Division of Thoracic Surgery, Department of Surgery, China Medical University Hospital, Taichung, Taiwan; i Surgical Intensive Care Unit, China Medical University Hospital, Taichung, Taiwan.

**Keywords:** acupuncture, medical costs, mortality, oral cancer, traditional Chinese medicine

## Abstract

Oral cancer is frequently associated with smoking, alcohol consumption, and betel quid chewing, which are common harmful behaviors observed in certain cohorts of the Taiwanese population. Some reports have explored the potential therapeutic effect of certain herbal remedies on cancer treatments and the outcomes thereof. However, supportive evidence regarding the specific use of traditional Chinese medicine (TCM) in oral cancer treatment is lacking and deserves further investigation. This study measured the use of TCM therapies for oral cancer in a Taiwanese population-based retrospective longitudinal cohort study. The Taiwan National Health Insurance Research Database was utilized to conduct this study. The study population was limited to oral cancer patients diagnosed between 2000 and 2009, which were followed up for at least 5 years. Therapeutic strategies investigated included acupuncture and the Chinese herbs and formula used. Additionally, the frequency of TCM treatment visits, total medical costs, and all-cause mortality were also analyzed. Between 2000 and 2009, a total of 951 patients were diagnosed with various oral cancers. 13.7% of the diagnosed patients utilized TCM treatment measures. The majority of the patients were males. The top 3 common single herbs used were *Xuán shēn (Radix Scrophulariae), Shí hú (Herba Dendrobii), and Mài mén dōng (Ophiopogon Japonicus*). Then, *Gān lù yǐn*, *Zhī bǎi dì huáng wán*, and *Sàn zhǒng kuì jiān tāng* were the most frequently used herbal formulas. The survival probability was higher in TCM users when compared to non-TCM users in 5- and 12-year all-cause mortality (*P* < .05). This study explored the use of TCM therapies in oral cancer patients and identified essential information regarding the specifics of conventional herbal medicine used, affiliated medical costs, survival probability, and common symptoms observed in Taiwanese oral cancer patients.

## 1. Introduction

In 2020, the prevalence of oral cancer was recorded at an incidence of 0.37 million new cases alongside a mortality value of 0.17 million.^[1]^ High risk behaviors observed in South Asian and Southeast Asian cohorts have given rise to an increased prevalence of etiological conditions prevailing in cancers of the head and neck.^[2]^ Given the popularity of the primary high risk behaviors such as smoking, alcohol consumption and betel quid chewing in these regions, the increased incidence of oral cancer is epidemiologically relevant.^[3,4]^ According to Xia et al,^[2]^ the greatest societal burden of oral cancer is observed in Pakistan, Taiwan, China, and India. Furthermore, the highest risk factor is attributed to cigarette smoking, followed closely by alcohol consumption and chewing tobacco.^[2]^ Subsequent evidence produced by Lee et al^[5]^ denotes the incidence of betel quid chewing to be of most relevance in regard to increased risk for oral cancer development. Gupta et al^[6]^ conducted a meta-analysis which revealed that the risk for oral cancer following the cessation of betel quid chewing remains high, and individuals are still at high-risk of cancer development within the first 10 years after cessation. Further, the incidence of alcohol consumption is directly affiliated with the increased risk of head and neck cancers in Asian populations.^[7]^ Lee et al,^[8]^ identified that an affiliated genetic mutation of ethanol-metabolizing genes, specifically the genotypes of alcohol dehydrogenase 1B and alcohol dehydrogenase H2, results in the poor overall survival of diagnosed head and neck cancer patients in addition to exponentially increasing the risk of cancer development. The human papilloma virus, which is widely accepted as an essential contributor in the development of cervical cancer, and is known to be commonly transmitted through sexual behavior, and was subsequently identified as an significant contributor in the incidence of oral cancer pathophysiology.^[9–11]^ Furthermore, the prevalence of several psychological disorders, such as depression, have been directly affiliated with the incidence of oral cancer, evidenced in a systematic review of the European population that indicated 28% of diagnosed oral cancer patients presented with comorbid symptoms of depression.^[12]^

Early diagnosis and clinically implemented oral cancer screening have been evidenced to provide suitable relief to both medical systems and patients alike as a beneficial and cost-effective strategy, although further research is required in this regard.^[13]^ Currently, evidence supports that education of the population facilitates improved understanding of the disease and its associated risks in addition to highlighting well-known risk factors, and all of which can aid in the prevention of oral cancer development.^[14]^ These functional approaches also provide an opportunity to decrease the burden of cost for oral cancer care and treatment with regard to both direct and indirect costs. In 2008, a financial report of head and neck cancer, which was inclusive of the oral cavity, pharynx, larynx, sinuses, and salivary glands cancers, detailed a total financial deficiency cost of up to US$8.5 billion in the United States of America and £255 million in the United Kingdom.^[15]^ Furthermore, cancer patients reported to be in metastatic or recurrent stages, and who underwent surgery within the first year, inclusive of follow-up periods, were representative of the highest cost and bore the highest financial burden.^[15]^ A systematic review similarly demonstrated the significant financial burden associated with the prevalence of oral cancer, specifically indicating an exponential increase in medical costs of later stage cancer.^[16]^

Common allopathic interventions for head and neck cancer include radiotherapy and chemotherapy, both of which inevitably result in a diverse array of negative side effects, such as oral mucositis.^[17]^ As an alternative treatment, curcumin administration, whether through oral administration and/or mouthwash application, demonstrates a positive ability to reduce the onset and severity of treatment toxicity, directing particular attention and the significant analgesic properties and pain management effects of this treatment.^[18]^ Moreover, oral cancer apoptosis, autophagy and mitochondria-dependent pathways have all exhibited activation in response to curcumin stimulation and support the therapeutic benefit of this alternative therapy.^[19–21]^ Furthermore, the daily consumption of Oolong tea was found to significantly reduce the risk head and neck cancer development.^[22]^ With regard to commonly used traditional Chinese medicine (TCM) herbal remedies, *Danshen* (*Radix Salviae Miltiorrhizae*) has reportedly demonstrated clear anti-inflammatory properties through tumor cell proliferation reduction via apoptotic cell activity and MAPK signaling decreases of oral cancer cells.^[23,24]^ A meta-analysis conducted by Bae et al,^[25]^ identified the herbal medicine exhibits the potential to increase natural killer cells in cancer patients. Furthermore, herbal medicine intervention demonstrates the potential to reduce multiple chemotherapy and radiotherapy induced side effects, such as xerostomia, gastrointestinal reactions, peripheral neuropathy, and pneumonitis.^[26,27]^ However, with regard to the combination of herbal medication, food, and allopathic drug administration in cancer patients, the importance of identifying and minimizing any food-drug toxicities should be emphasized.^[28]^

The objectives of this study were to investigate the use of TCM among oral cancer patients. This was investigated through a retrospective longitudinal cohort study conducted from 2000 to 2009.

## 2. Methods

### 2.1. Data source

The data of this retrospective longitudinal cohort study were obtained from the Taiwan National Health Insurance Research Database (NHIRD) and the Longitudinal Health Insurance Database 2000. The outpatient, hospital admission, medication, and treatment data of 1 million randomly selected subjects (approximately 5% of the total population of Taiwan) were used. The study was approved by the Institutional Review Board of China Medical University (CMUH104-REC2-115-CR7) (see Supplementary materials, Supplemental Digital Content, http://links.lww.com/MD/xxx). As the database does not include identifying patient details, a waiver of the consent requirement was granted by the Institutional Review Board.

### 2.2. Study population

The patients who were diagnosed with oral cancer between 2000 and 2009 comprised the primary study population. Oral cancer patients were defined in accordance with the diagnostic code of the International Classification of Diseases, Ninth Revision, Clinical Modification (ICD-9-CM) 140-145. Patients aged younger than 20 years of age were excluded. Patients were divided according to the treatments received. The first group was inclusive of a non-TCM group incorporating patients who did not receive TCM throughout the stated study period. The second group was inclusive of the TCM group, incorporating patients that received TCM interventions throughout the stated study period. A follow-up period of at least 5 years was stated.

### 2.3. Interventions

The demographic information of patients that received western medicine intervention during the study period was collected according to the following ICD-9-CM codes: antineoplastic V58.11; chemotherapy V66.2; systemic chemotherapy 99.25; radiotherapy V66.1; stem cells V42.82, V42.3, V42.9; and encounter for antineoplastic immunotherapy V58.12. Furthermore, the demographics of patients that received surgery during the study period was collected according to the following ICD-9-CM codes: 27.1, 27.31, 27.32, 27.41, 27.42, 27.43, 27.49, 27.51, 27.52, 27.53, 27.54, 27.55, 27.56, 27.57, 27.57, 27.59, 27.6, 27.61, 27.62, 27.63, 27.64, 27.69, 27.7, 27.71, 27.72, 27.73, 27.79, 27.9, 27.91, 27.92, and 27.99. For the TCM group, patient demographics were collected according to the following acupuncture intervention ICD-9-CM codes: manual acupuncture B41, B42, B80-B84, B90-B94, P27041, P31103, P32103, and P33031; electro-acupuncture B43, B44, B86-89, and P33032; and complex acupuncture B45 and B46.

### 2.4. Sample size

All oral cancer patients recorded in Taiwan between 2000 and 2009, who were registered under the above-mentioned ICD-9-CM codes were included in this study.

## 3. Outcome measurements

### 3.1. Symptoms

We investigated specific symptoms that required medical attention 5 years after the diagnosis of cancer. The included symptoms were recorded according to the following ICD-9-CM codes: pain 780.96; 784.1; dyspnea and respiratory abnormalities 786.0; malnutrition 263.9, 579.3, 783.2, 783.3, 783.7, 995.84, and V85.0; cachexia 260, 261, 262, 263.0-263.1, 263.3-263.9, and 799.4; oral soft tissues symptoms 528.2, 528.9, and 784.2; neoplasm 140-146, 149, 173.00, and 173.09; digestive system 525.8, 527.7, 528.6, 528.79, 530.1, 531-537, 564, 578.1, 787.01, and 787.91; injury 840-848; dysarthria 784.51; swelling, mass, or lump in head and neck 784.2; and other symptoms 780.50, 780.52, 780.60, 780.61, 780.96, 780.99, 781.99, 782.9, 784.0, 784.1, 784.99, 785.9, 786.00, 786.2, 786.50, 786.9, 787.02, 787.03, 787.20, 787.21, 787.22, 787.23, 787.24, 787.29, 789.00, 789.07, 789.09, 789.7, 789.9, 788.30, 788.99, and 796.9.

### 3.2. Medical cost

The medical costs of individual patients were recorded 1-year post-diagnosis. The out-patient visits costs were inclusive of diagnosis, treatment, and drug cost. The hospitalization cost included diagnosis, drug administration, radiotherapy, other therapies, and surgery.

### 3.3. Chinese herbs and formulas used

The 10 most common Chinese herbs and the 10 most common Chinese formulas used were investigated.

### 3.4. Mortality

All-cause mortality and survival probability were measured at 5- and 12-year follow-up.

### 3.5. Statistical analysis

The distribution of patient characteristics across different treatment groups was examined by a Chi-square test and the Student *t* test. The odds ratio (OR) with 95% confidence interval (CI) was estimated by the logistic model for symptoms after 5 years, and was adjusted according to sex, age, urbanization, occupation, and residential location variables. The medical cost between 2 groups was compared by the Student *t* test. The Kaplan–Meier method was applied to obtain the all-cause mortality and survival curves, and tested by the log-rank test. The all-cause mortality hazard ration (HR) and 95% CI was adjusted by age, gender, chronic kidney disease (CKD), and diabetes. All statistical analyses were performed by SAS (version 9.4; SAS Institute, Inc., Cary, NC) and a 2-tailed *P* value < .05 was considered to be statistically significant. The graph was plotted using software R (version 3.6.3).

## 4. Results

During the period of 2000 to 2009, a total of 951 patients were diagnosed with oral cancer. Of these patients, 821 did not use TCM (86.3%), and a further 130 patients used TCM (13.7%). Out of the TCM users, a total of 10 patients also used acupuncture (7.7%).

Female patients 20 (15.4%) were more willing to receive TCM in addition to western medicine versus only 77 (9.4%) of the females in the non-TCM group. There were more males 744 (90.6%) in the non-TCM group versus 110 (84.6%) of the TCM users (*P* = .04). The majority of the patients in both groups were aged between 50 and 64 years old. Interestingly, the number of patients in the TCM group older than 65 years old were fewer than the non-TCM group (14.6% vs 22%), although the difference was not significant. Patients who received western medicine only had an older mean age (54.08 ± 12.9), and patients in TCM group had a younger mean age (51.74 ± 11.7). There are no significant differences in patients’ living urbanization, occupation, and monthly income. TCM users tend to live in the center of Taiwan, with 43.1% versus 28.6% in the non-TCM group (*P* = .02). Medical centers were also observed to be the major care provision facility used in both groups (97.1% vs 96.2%). With regard to comorbidities, there were significantly fewer diabetic cases recorded in the TCM user group 16.9% versus 26.6% in the non-TCM group (*P* = .02). Furthermore, there were also significantly less incidences of CKD comorbidity in the TCM group, at 5.4% versus 11.8% in the non-TCM group (*P* = .03). With regard to the other comorbidities, the non-significant results demonstrated that the TCM group also had less incidences of hypertension, congestive heart failure, stroke and anemia-related diagnoses. The average number of TCM visits was recorded as 12.4 ± 21.2 over 9.73 ± 16.1 months (see Table 1).

**Table 1:** Patients demographics.

Characteristics	Non-TCM (N = 821)		TCM (N = 130)		*P* value
	n	%	n	%	
Sex					.04
Female	77	9.4	20	15.4	
Male	744	90.6	110	84.6	
Age, yr					.10
≤49	304	37.0	58	44.6	
50–64	336	40.9	53	40.8	
≥65	181	22.0	19	14.6	
Mean age (SD)	54.08	12.9	51.74	11.7	.05
Monthly income (NTD)					.16
<15,000	206	25.1	25	19.2	
15,000–20,000	349	42.5	53	40.8	
>20,000	266	32.4	52	40.0	
Urbanization					.28
Low	373	45.4	68	52.3	
Medium	261	31.8	39	30.0	
High	187	22.8	23	17.7	
Occupation					.17
Office Workers	325	39.6	61	46.9	
Agricultural Workers	398	48.5	59	45.4	
Other	98	11.9	10	7.7	
Residential location					.02
Northern	273	33.3	36	27.7	
Central	235	28.6	56	43.1	
Southern	289	35.2	34	26.2	
Eastern	23	2.8	4	3.1	
Other	1	0.1	0	0.0	
Service providers					.27
Medical Center	797	97.1	125	96.2	
Regional Hospital	17	2.1	5	3.8	
District Hospital	7	0.9	0	0.0	
Comorbidities					
Hypertension	368	44.8	50	38.5	.17
Diabetes	218	26.6	22	16.9	.02
CHF	68	8.3	7	5.8	.25
Stroke	119	14.5	13	10.0	.17
COPD	185	22.5	31	23.8	.74
Cirrhosis	8	1.0	0	0.0	.26
CKD	97	11.8	7	5.4	.03
Anemia	146	17.8	17	13.1	.19
Malignant neoplasm of bronchus and lung	1	0.1	1	0.8	.13
CCI					.26
0	813	99.0	130	100.0	
1	0	0.0	0	0.0	
≥2	8	1.0	0	0.0	
Treatment					
Western therapy	283	34.5	56	43.1	.06
Oral cancer surgery	89	10.8	16	12.3	.62
Number of visits, mean (SD)			12.4	21.2	
Length of TCM use (mo) months, mean (SD)			9.73	16.1	

The table represents the general characteristics recorded in the included cohort of oral cancer patients. Data are presented as mean, SD, number of patients (N), and percentage value (%). Significant levels are considered as *P* values of <.05.

CCI = Charlson Comorbidity Index, CHF = congestive heart failure, CKD = chronic kidney disease, COPD = chronic obstructive pulmonary disease, SD = standard deviation.

After 5 years from the initial cancer diagnosis, patients in the TCM group had significantly more symptoms of pain (OR = 2.50; 95% CI = 1.02, 6.14; *P* = .05) and oral soft tissues symptoms (OR = 2.68; 95% CI = 1.44, 4.96; *P* = .002) (Table 2).

**Table 2:** Common symptoms leading to medical consultation 5 years post-diagnosis.

Symptoms after 5 years	Non-TCM (N = 821)			TCM (N = 130)					
	N	%	OR	N	%	cOR (95% CI)	*P* value	aOR (95% CI)	*P* value
Pain	20	2.44	1.00 (reference)	8	6.15	2.63 (1.13, 6.09)*	.02	2.50 (1.02, 6.14)*	.05
Dyspnea and respiratory abnormalities	42	5.12	1.00 (reference)	7	5.38	1.06 (0.46, 2.40)	.90	1.02 (0.43, 2.43)	.96
Malnutrition	29	3.53	1.00 (reference)	5	3.85	1.09 (0.42, 2.87)	.86	0.97 (0.36, 2.63)	.95
Cachexia	50	6.09	1.00 (reference)	11	8.46	1.43 (0.72, 2.82)	.31	1.40 (0.69, 2.84)	.35
Oral soft tissues symptoms	51	6.21	1.00 (reference)	18	13.85	2.43 (1.37, 4.30)**	.002	2.68 (1.44, 4.96)**	.002
Neoplasms	0	0.00	1.00 (reference)	0	0.00				
Alcohol related	15	1.83	1.00 (reference)	2	1.54	0.84 (0.19, 3.71)	.82	0.60 (0.12, 2.94)	.53
Digestive system disease	89	10.8	1.00 (reference)	16	12.3	1.15 (0.65, 2.04)	.62	1.10 (0.60, 2.02)	.75
Injury	53	6.46	1.00 (reference)	8	6.15	0.95 (0.44, 2.05)	.90	0.90 (0.40, 2.01)	.80
Other	131	16.0	1.00 (reference)	27	20.8	1.38 (0.87, 2.19)	.17	1.33 (0.81, 2.17)	.26

Data are presented as number of patients (N) and % thereof, OR, cOR, aOR, and CI.

aOR = adjusted odds ratio, CI = confident interval, cOR = crude odds ratios, OR = odds ratios.

* *P* value < .05.

** *P* value < .01.

With regard to the medical cost, after 1-year post-diagnosis, it was observed that a significantly higher cost of diagnosis in the outpatient visits of the TCM group was recorded, when compared to the non-TCM group in New Taiwan Dollar (7929 ± 5717 TWD vs 6195 ± 5879 TWD, respectively; *P* = .002). However, no significant differences in the overall total cost of both outpatient visits and hospitalization between the 2 groups was observed (*P* > .05) (Table 3).

**Table 3:** Total medical cost of oral cancer patients at 1-year post-diagnosis follow up.

Medical cost after 1 year	Non-TCM (N = 821)		TCM (N = 130)		*P* value
	Mean	SD	Mean	SD	
Outpatient visit					
Diagnosis	6195	5879	7929	5717	.002
Treatment	57,524	101,598	58,996	80,425	.86
Drug	38,627	88,746	61,276	88,145	.11
Total	76,673	113,213	85,945	93,895	.38
Hospitalization					
Diagnosis	16,268	13,198	17,061	13,030	.62
Drug therapy	69,968	100,696	75,690	10,857	.67
Radiotherapy	44,014	78,165	454,831	75,257	.88
Other therapies	13,074	19,261	11,210	17,185	.41
Surgery	58,380	62,322	79,069	77,546	.14
Total	319,706	314,347	339,184	323,449	.62

Data are presented in mean and SD.

SD = standard deviation, TCM = traditional Chinese medicine.

Statistical significance is considered as a *P* value of <.05.

Table 4 records the top ten single herbs and herbal formulas used by oral cancer patients in the TCM-group. Of these, the top 3 common single herbs were玄參 Xuán shēn (Radix Scrophulariae), 石斛 Shí hú (Herba Dendrobii), and 麥門冬 Mài mén dōng (Ophiopogon Japonicus). Thereafter, 甘露飲 Gān lù yǐn, 知柏地黃丸 Zhī bǎi dì huáng wán, and 散腫潰堅湯 Sàn zhǒng kuì jiān tāng were the most frequently used herbal formulas (Table 4).

**Table 4:** Top 10 traditional Chinese single herbs and herbal formulas used.

	Single herbs	Herbal formulas
1	玄參 Xuán shēn (Radix Scrophulariae)	甘露飲 Gān lù yǐn
2	石斛 Shí hú (Herba Dendrobii)	知柏地黃丸 Zhī bǎi dì huáng wán
3	麥門冬 Mài mén dōng (Ophiopogon Japonicus)	散腫潰堅湯 Sàn zhǒng kuì jiān tāng
4	山查 Shān chá (Fructus Crataegi)	歸脾湯 Guī pí tāng
5	川芎 Chuān xiōng (Rhizoma Ligustici Wallichii)	一貫煎 Yī guàn jiān
6	半夏 Bàn xià (Rhizoma Pinelliae Preparatum)	十六味流氣飲 Shí liù wèi liú qì yǐn
7	白朮 Bái zhú (Rhizoma Atractylodis Macrocephalae)	加味逍遙散 Jiā wèi xiāo yáo sàn
8	合歡皮 Hé huān pí (Cortex Albizziae)	平胃散 Píng wèi sàn
9	地骨皮 Di gǔ pí (Cortex Lycii)	甘麥大棗湯 Gān mài dà zǎo tāng
10	百合 Bǎihé (Bulbus Lilii)	生脈飲 Shēng mài yǐn

The numbers 1–10 represent the order of the most common (1) to the least common (10).

**Figure 1.**
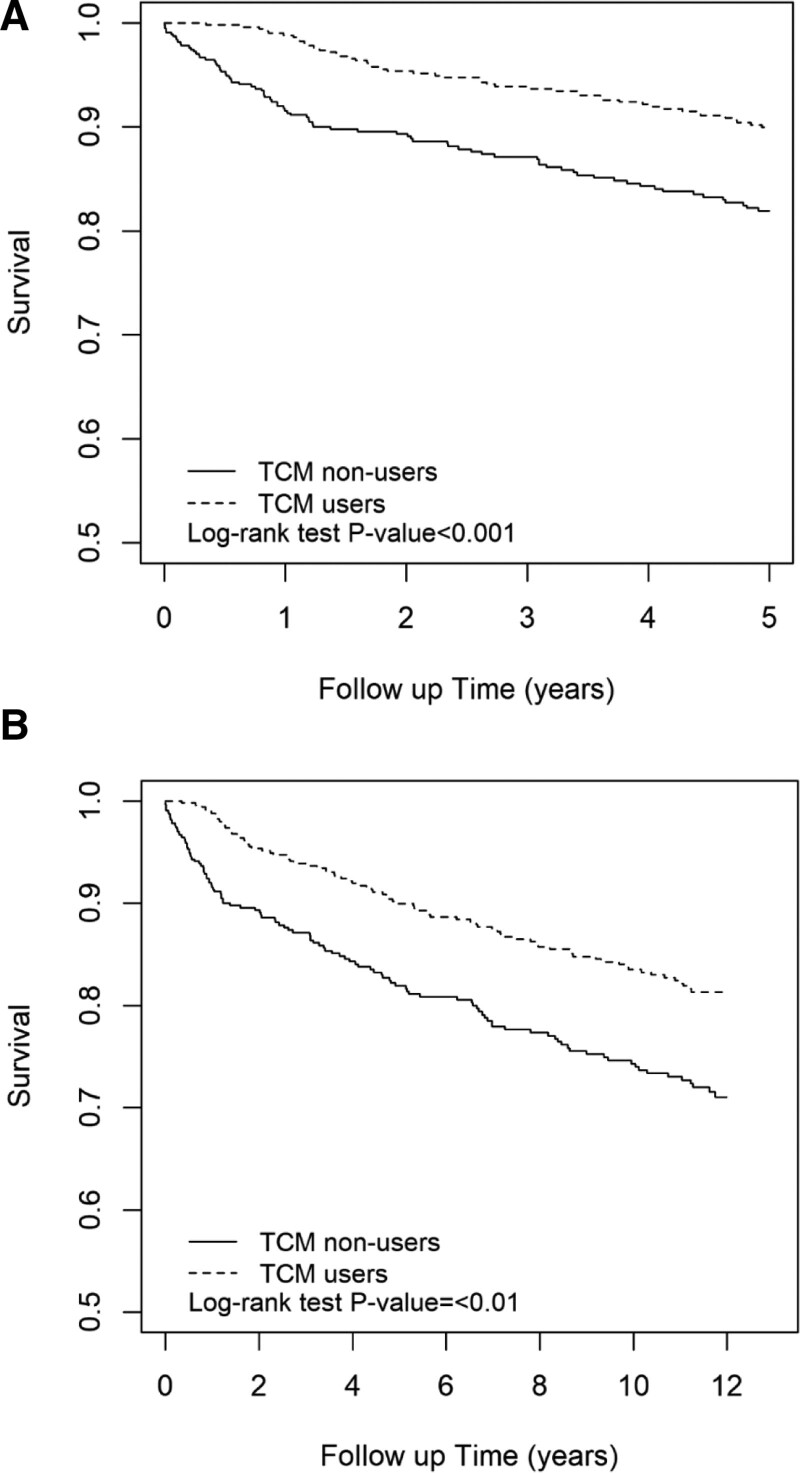
(A) Graphical representation of the 5 years all-cause mortality in both groups. (B) Graphical representation of the 5 years all-cause mortality in both groups. Significant levels are presented as a *P* value < .05.

Lastly, as depicted in Figure 1 and Table 5, the TCM group demonstrated a significantly higher 5-year survival measurement when compared to the non-TCM users (HR = 0.57, 95% CI = 0.40, 0.82; log-rank test *P* = .001). The findings also show that TCM users had a significantly higher 12-year survival measurement when compared to the non-TCM users (HR = 0.64, 95% CI = 0.48, 0.85; log-rank test *P* = .01). The all-cause mortality CI was adjusted by age, gender, CKD and diabetes comorbidities. This finding provides evidence for the overall benefit of TCM therapy with regard to improved survival outcomes and all-cause mortality (Fig. 1).

**Table 5:** The 5- and 12-year all-cause mortality analysis.

	5-year all-cause mortality		12-year all-cause mortality	
	Adjusted HR	95% CI	Adjusted HR	95% CI
TCM non-users	1.00	Reference	1.00	Reference
TCM users	0.57	0.40, 0.82**	0.64	0.48, 0.85**

The table represents the risk for all-cause mortality in TCM users compared to non-TCM users. The HR and CI were adjusted by age, gender, CKD, and diabetes comorbidities.

CKD = chronic kidney diseases, CI = confidence interval, HR = hazard ratio, TCM = traditional Chinese medicine.

**P value < .01.

## 5. Discussion

This research work is a retrospective longitudinal cohort study investigating the use of TCM in oral cancers. The study findings include improved 5- and 12-year survival outcomes for individuals who utilize TCM therapies, although patients in the TCM group experienced more pain and oral soft tissues symptoms. The most commonly used single herb and formula were 玄參 Xuán shēn (Radix Scrophulariae) and 甘露飲 Gān lù yǐn, respectively. The total medical costs recorded 1-year post-diagnosis were similar between the 2 groups. Since a majority of the included patients utilized conventional treatment approaches, the general characteristics of the smaller TCM using cohort were identified according to the use of herbal therapies as well as acupuncture treatment. The male to female ratio of our study supported the proclivity of increased oral cancer prevalence in males.^[29]^

Several risk associations affiliated with oral cancer development have been identified, and include certain genetic variants associated with alcohol metabolism, nicotine metabolism, and associated DNA repair.^[30,31]^ Unfortunately, this study was unable to include the measurements of patient alcohol consumption due to the stringent NHIRD database limitations. Interestingly, this study identified a very low number of patients seeking treatment for alcohol related symptoms after 5 years. This work has identified potential genetic-environmental risk interactions observed in incidences of oral cancer, alongside increased incidences of diabetes and CKD comorbidities in the conventional western medicine treatment group. The pathogenic involvement of these disease conditions has been identified in constituting primary factors in the affiliated disease development, and in conjunction with relatively strong hereditary risk associations, the prevalence of oral cancer is therefore of greater concern in these populations.^[32]^ The primary finding of this study is that of an increased survival outcome in the group using TCM therapies. A possible explanation regarding this particular observation can be associated with the therapeutic effects of the most common herbal medicine formula used in this study; *Gan Lu Yin*. This formula consists of active ingredients (*Rehmannia glutinosa, Liriope spicata (Thunb.) Lour, Eriobotrya japonica (Thunb.) Lindl, Citrus sinensis Osbeck, Glycyrrhiza uralensis Fisch, Artemisia capillaris Thunb, Dendrobium nobile Lind., and Scutellaria baicalensis Georgi.*) that have been evidenced to not only diminish the MAPK signaling of oral cancer cells, but also reduce TNF–α secretion.^[33]^ The second most commonly used formula, *Zhī bǎi dì huáng wán*, is a formula that is frequently prescribed for a TCM diagnosis of yin depletion, and has been clinically evidenced to have associated with a reduced risk of breast cancer in a cohort of female patients diagnosed with type 2 diabetes.^[34]^ The formula, *Guī pí tang*, is often prescribed for a diagnosis of anemia and has been associated with improved survival in patients diagnosed with acute myeloid leukemia.^[35]^ Furthermore, 2 of the top 3 single herbs used in this study, namely *玄參 Xuán shēn (Radix Scrophulariae) and麥門冬 Mài mén dōng (Ophiopogon Japonicus*), were associated with a lower cancer-related mortality risk in patients with advanced nasopharyngeal cancer.^[36]^ Additionally, Yang et al,^[37]^ conducted a meta-analysis that evidenced instances of combined TCM and conventional medicine therapy that can increase the overall survival in hepatocellular carcinoma patients. Furthermore, since the average number of TCM visits and the length of TCM use were relatively high in patients of this study (12.4 visits over 9.73 months), the results suggest that regular intervals of treatment over a long period of time are effective in maintaining a relative degree of homeostasis, and this can be readily observed in multiple current therapeutic strategies.^[38]^ This positive outcome may therefore be associated with the increase in survival observed in the TCM group. The number of patients in the non-TCM group that were reported to have diabetic and CKD comorbidities was higher than that of TCM group. However, in the survival analysis we adjusted the HR to match those comorbidities along with imbalances in patients’ age and sex. Nevertheless, this study did not include cancer staging data, as such data are unavailable in the NHIRD database. Cancer staging data, along with specific cancer related morality rates are both important factors that require further investigation in future studies.

The increased number of doctor visits for pain and oral soft tissue symptoms recorded in the TCM group may serve to explain the general opinion of patients that TCM therapies can be beneficial for pain and oral soft tissue symptoms, which is correspondingly supported by current literature.^[27,39–41]^ Further, while the cost of outpatient consultations adds to the increased financial expense over time, no significance in the financial burden was discovered. Additionally, Taiwan is renowned for the affordable and accessible healthcare infrastructure that is available to the public, to which end the comprehensive system serves to provide relevant information regarding accurate cost of cover for TCM therapy as it is included as a public health service.^[42]^ The medical cost encountered by TCM users might be considerably higher in other locations across the world where TCM is not covered by the national health insurance and instead forms part of private health costs.^[43]^

Acupuncture was not widely used as an oral cancer treatment in this study. Despite evidence that acupuncture can benefit cancer patients in terms of reducing chemotherapy-induced side effects and despite the low cost of acupuncture treatments in Taiwan.^[27]^

### 5.1. Limitations

In this study, there were serval limitations. The study sample size serves as a limitation since only 951 patients were enrolled in this study. The study did not use randomization or matching of the patients in both groups as the number of patients was not sufficient. Data on important confounding factors for oral cancer patients, such as smoking, alcohol consumption, and betel quid chewing were not available on the NHIRD system. Data on cancer staging were also not available in the NHIRD system. Only data on all-cause mortality were available in the NHIRD system and that did not allow for a specific analysis of cancer-related mortality.

## 6. Conclusion

This study provides current, clinically applicable information on the use of TCM in oral cancer patients in Taiwan. The study includes trends of Chinese herbal medicine use as well as common symptoms preceding and resulting in medical attention. The study also provides important data on the affiliated medical costs and estimations of the 5- and 12-year survival for oral cancer patients in Taiwan. The relevance of these findings provides valuable insight concerning oral cancer treatment trends presents important evidence regarding the combination therapy of TCM in cancer treatment. This study can assist TCM professionals and health care providers in the development of complementary guidelines for treating oral cancer patients.

## Author contributions

**Conceptualization:** Eyal Ben-Arie, Yu-Chen Lee, Pei-Yu Kao, Wen-Chao Ho, Hei-Tung Yip.

**Data curation:** Hei-Tung Yip.

**Formal analysis:** Hei-Tung Yip.

**Funding acquisition:** Hei-Tung Yip.

**Investigation:** Eyal Ben-Arie, Yu-Chen Lee, Pei-Yu Kao, Wen-Chao Ho, Hei-Tung Yip.

**Methodology:** Eyal Ben-Arie, Yu-Chen Lee, Pei-Yu Kao, Wen-Chao Ho, Hei-Tung Yip, Gil Ton.

**Project administration:** Eyal Ben-Arie, Yu-Chen Lee, Pei-Yu Kao.

**Resources:** Eyal Ben-Arie, Yu-Chen Lee, Pei-Yu Kao.

**Software:** Hei-Tung Yip.

**Supervision:** Yu-Chen Lee, Pei-Yu Kao.

**Validation:** Chanya Inprasit, Bernice Lottering.

**Visualization:** Eyal Ben-Arie, Hei-Tung Yip.

**Writing – original draft:** Eyal Ben-Arie, Chanya Inprasit, Bernice Lottering.

**Writing – review & editing:** Eyal Ben-Arie, Yu-Chen Lee, Pei-Yu Kao, Wen-Chao Ho, Hei-Tung Yip, Chanya Inprasit, Bernice Lottering, Gil Ton.

## Correction

The funding information “Taiwan Ministry of Health and Welfare Taiwan Ministry of Health and Welfare (MOHW111-CMAP-M-113-112103)” has been corrected to “Taiwan Ministry of Health and Welfare (MOHW111-CMAP-M-113-112101).”
